# Occurrence–regression–recurrence of hepatocellular carcinoma without any intervention: A case report

**DOI:** 10.3389/fsurg.2022.972446

**Published:** 2022-09-12

**Authors:** Weinan Li, Yongfu Xiong, Xia Shu, Jingdong Li

**Affiliations:** ^1^ Department of Hepatobiliary Surgery, Affiliated Hospital of North Sichuan Medical College, Nanchong, China; ^2^ Department of Gastroenterology, First People’s Hospital of Longquanyi District, Chengdu, China

**Keywords:** hepatocellular carcinoma, spontaneous regression, case report, ischemia, immune response

## Abstract

**Background:**

Spontaneous regression of primary liver cancer is a rare event, and currently the exact pathogenesis of spontaneous tumor regression remains unclear.

**Case description:**

Clinical information was collected from a patient with spontaneous regression of liver cancer at our center. The patient was a 41-year-old male. He was admitted to the hospital on 3 May 2019, due to aversion to fatty or greasy food, anorexia, loss of appetite, and abdominal distension. Laboratory examination results included hepatitis B surface antigen positivity, hepatitis B e antigen positivity, and hepatitis B core antibody positivity and tumor marker levels of alpha-fetoprotein 142,938.20 µg/L, abnormal prothrombin 4,599.91 mAU/ml, and carbohydrate antigen 19–9 82.05 U/ml. Upper abdominal enhanced computed tomography indicated right hepatocellular carcinoma with portal vein tumor thrombus formation. The patient declined any treatment. The tumor in the right lobe of the liver completely regressed after 1 year, and the patient is still undergoing follow-up.

**Conclusions:**

We encountered a hepatocellular carcinoma patient who underwent spontaneous regression, but the exact pathogenesis remains unknown. Understanding the pathogenesis of spontaneous regression of hepatocellular carcinoma has the potential to contribute to the development of an effective treatment for hepatocellular carcinoma.

## Introduction

Spontaneous regression of malignant tumors refers to their partial or complete disappearance without any treatment (1), and it is very rare. The incidence rate is approximately 1 in 6–10 million cases (2). Spontaneous regression of malignant tumors has been reported for various tumor types, including renal cell carcinoma (3), neuroblastoma (4), malignant melanoma (5), malignant lymphoma (6), and leukemia (7).

Hepatocellular carcinoma (HCC) is a liver malignancy with a very high incidence (8). Surgical treatment is currently the preferred treatment (9). Because early-stage HCC commonly lacks specific clinical symptoms, it is often at a late stage when discovered (10). Therefore, the opportunity for surgical treatment is lost, and long-term survival is low (11). It is thus necessary to develop novel therapies for advanced HCC. Previous studies have focused on the investigation of HCC pathogenesis for therapy, rather than mechanisms involved in spontaneous regression.

Herein, we describe the case of a patient who underwent complete spontaneous regression of HCC without any intervention. No more than 20 cases of spontaneous regression of HCC have been reported, and the exact mechanism of spontaneous regression of tumors remains unclear. Investigation of the mechanisms involved in spontaneous regression of HCC may be a fruitful line of inquiry with respect to HCC therapy.

## Case presentation

**Figure 1:**
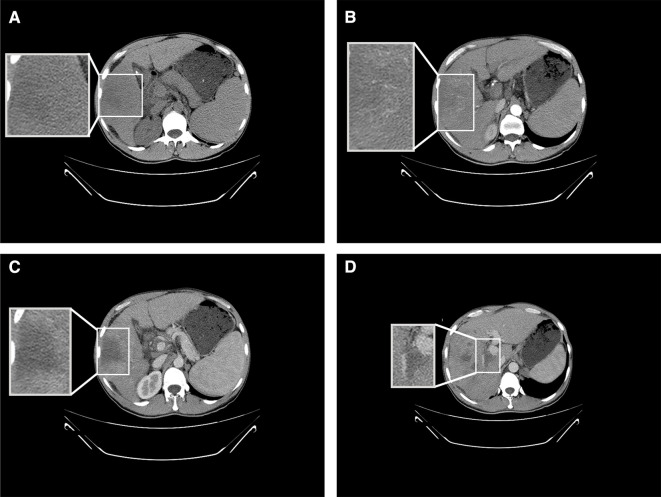
Results of CT conducted on 3 May 2019. (**A**) Upper abdominal CT depicting a low-density shadow in the right lobe of the liver. (**B**) Enhanced CT showing uneven enhancement of the shadow in the right lobe of the liver at the artery stage. (**C**) Enhanced CT indicating fast enhancement regression of the shadow in the right lobe of the liver at the vein stage. (**D**) Enhanced CT depicting tumor thrombus formation in the portal vein.

The patient was a 41-year-old male. He was admitted to the hospital on 3 May 2019 due to aversion to fatty or greasy food, anorexia, loss of appetite, and abdominal distension. He stated that he did not have a history of hepatitis B, hepatitis C, smoking, or long-term drinking. Physical examination did not reveal any abdominal tenderness, rebound tenderness, or muscle tension, and the liver and spleen were not palpable under the ribs. The results of blood analysis included total white blood cell count of 2.63 × 10^9^/L, hemoglobin of 112 g/L, and platelet count of 33 × 10^9^/L (Supplementary Table S1). Liver function test results included aspartate aminotransferase (AST) of 95 U/L, alanine aminotransferase (ALT) of 41 U/L, alkaline phosphatase (ALP) of 105 U/L, albumin of 34.2 g/L, total bilirubin of 37.3 μmol/L, direct bilirubin of 13.7 μmol/L, and indirect bilirubin of 23.6 μmol/L (Supplementary Table S1). Hepatitis B-associated test results included hepatitis B surface antigen (HBsAg) positivity, hepatitis B e antigen positivity, and hepatitis B core antibody positivity. Tumor marker test results included alpha-fetoprotein (AFP) of 142,938.20 μg/L, abnormal prothrombin of 4,599.91 mAU/ml, and carbohydrate antigen of 19–9 82.05 U/ml. The AFP in this patient was far in excess of the normal range. Imaging was then performed in-house. Upper abdominal computed tomography (CT) revealed a giant mass with dimensions of approximately 6.2 × 5.5 cm in the right lobe of the liver (Figure 1A). The enhanced scan exhibited the characteristics of fast-in and fast-out enhancement (Figures 1B,C). HCC was therefore considered, and tumor thrombus formation was noted in the portal vein (Figure 1D). Given the combination of AFP level and imaging information, the patient met the standard for the clinical diagnosis of HCC (12). Interventional, immune, and molecular targeted combined therapy was offered to the patient, but he declined any treatment.

**Figure 2:**
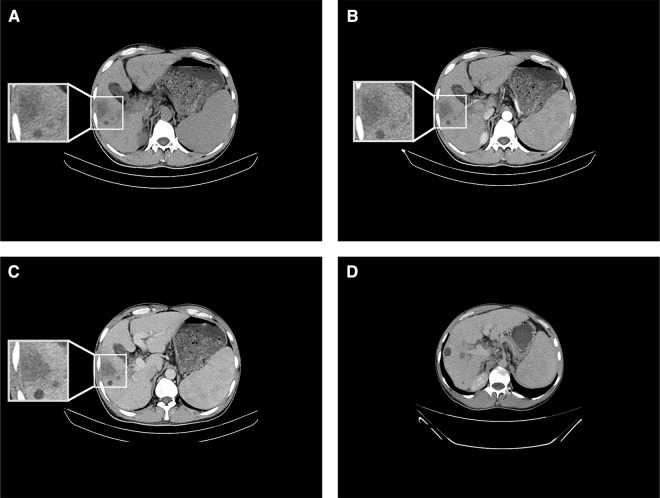
Results of CT conducted on 23 July 2019. (**A**) Upper abdominal CT showing a low-density shadow in the right lobe of the liver. (**B**) Enhanced CT showing no enhancement of the shadow in the right lobe of the liver at the artery stage. (**C**) Enhanced CT indicating no enhancement in the shadow in the right lobe of the liver at the vein stage. (**D**) Enhanced CT depicting portal vein tumor thrombus regression at the vein stage.

On 23 July 2019, the patient revisited our department for re-examination. The results of routine blood tests included total white blood cell count of 2.87 × 10^9^/L, hemoglobin of 143 g/L, and platelet count of 37 × 10^9^/L (Supplementary Table S2). Liver function test results included AST of 47 U/L, ALT of 49 U/L, ALP of 91 U/L, albumin of 40.9 g/L, total bilirubin of 27.5 μmol/L, direct bilirubin of 6.9 μmol/L, and indirect bilirubin of 20.6 μmol/L (Supplementary Table S2). Tumor marker results included dramatic reductions in AFP (to 91.60 μg/L) and abnormal prothrombin level (to 14.33 mAU/ml) and a comparatively modest reduction in carbohydrate antigen of 19–9 (to 38.93 U/ml). In upper abdominal CT, the mass lesion in the right lobe of the liver was not enhanced in either the artery stage or the vein stage; thus, the low-density shadow without enhancement at both the artery stage and the vein stage at the primary tumor location was considered to be a liquefied necrotic zone after spontaneous tumor regression (Figures 2A–C). The portal vein tumor thrombus had significantly diminished (Figure 2D). In conclusion, the mass was replaced by liquefaction necrosis zones, and AFP and abnormal prothrombin levels were dramatically reduced to within normal ranges, indicating that the primary tumor had diminished without any therapy.

**Figure 3:**
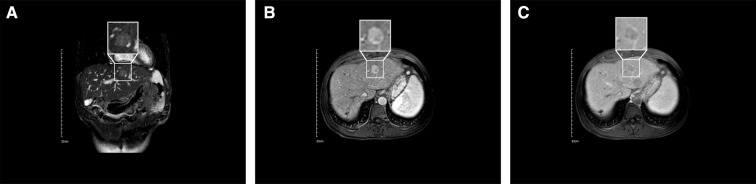
Results of MRI conducted on 11 January 2021. (**A**) Coronal MRI depicting a low-signal nodule in the left lobe of the liver. (**B**) Enhanced MRI depicting a high-signal nodule in the left lobe of the liver at the artery stage. (**C**) Enhanced MRI indicating fast enhancement regression of the nodule in the left lobe of the liver at the vein stage.

**Figure 4:**
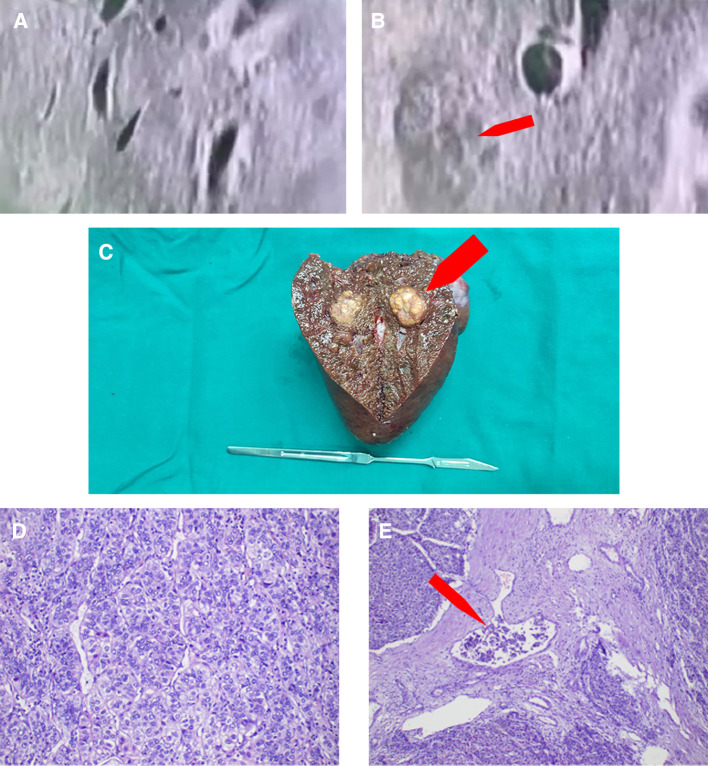
Intraoperative and postoperative examination results. (**A**) Intraoperative ultrasound depicting a complete regression of the original tumor. (**B)** Intraoperative ultrasound depicting a new tumor in the left lobe of the liver. (**C**) Gross postoperative gray–yellow solid nodule specimen. (**D**) Pathological examination showing a moderately differentiated hepatocellular carcinoma. (**E**) Pathological examination showing microvascular invasion in the tumor.

On 11 January 2021, upper abdominal magnetic resonance imaging (MRI) depicted no obvious space-occupying lesion at the former location of the primary tumor but a new space-occupying lesion in the left lateral lobe of the liver (Figure 3A). The enhanced scan exhibited the characteristics of fast-in and fast-out enhancement (Figures 3B,C). Routine blood test results included total white blood cell count of 3.17 × 10^9^/L, hemoglobin of 157 g/L, and platelet count of 31 × 10^9^/L (Supplementary Table S3). Liver function test results included AST of 37 U/L, ALT of 26 U/L, ALP of 67 U/L, albumin of 46.2 g/L, total bilirubin of 33.3 μmol/L, direct bilirubin of 11.7 μmol/L, and indirect bilirubin of 21.6 μmol/L (Supplementary Table S3). Tumor marker results included AFP of 860.20 μg/L and abnormal prothrombin of 35.83 mAU/ml. Based on the imaging information and tumor marker data, HCC was again diagnosed, but it was not clear whether the newly detected entity was a new tumor or a metastatic growth derived from the original tumor. Laparoscopic left hemihepatectomy was performed on 3 March 2021, and intraoperative ultrasound indicated no space-occupying lesion in the location of the original tumor but a space-occupying lesion in the left medial lobe of the liver. Postoperative gross specimen analysis revealed a gray–yellow solid nodule approximately 2.5 × 1.7 × 1.2 cm in size in the left medial lobe of the liver (Figure 4C), and pathological examination indicated moderately differentiated HCC with microvascular invasion (Figures 4D,E). The patient is still undergoing follow-up.

## Discussion and conclusion

The patient exhibited HBsAg positivity and significantly increased AFP and abnormal prothrombin levels. Upper abdominal CT depicted obvious imaging characteristics of HCC. Based on these observations, the patient met the standard for a clinical diagnosis of HCC combined with intrahepatic metastasis. In other words, the patient was suffering from late-stage HCC. Given the late stage of liver cancer, the opportunity for surgical treatment had been missed. In addition, the patient refused any treatment. During follow-up, the patient returned for a re-examination. Re-examination revealed that AFP and abnormal prothrombin levels significantly decreased, and enhanced abdominal CT depicted a low-density shadow at the location of the original tumor that was not enhanced in either the arterial stage or the vein stage. Based on these observations, the low-density shadow was considered to be a liquefaction necrosis area after spontaneous tumor regression. The tumor in the right lobe of the liver had regressed completely by 22 January 2021, but liver cancer foci were present in the left lobe, and the patient agreed for surgery at that time. During surgery, the liver was investigated *via* laparoscopy and intraoperative ultrasound. The findings only included space-occupying lesions in the left lobe of the liver, and the original cancer foci in the right lobe regressed completely. In conclusion, herein, we have described an interesting case of a patient who exhibited occurrence–regression–recurrence of HCC without any intervention.

Spontaneous regression of HCC was first reported by Cole and Everson (1) in 1956. Since then, complete spontaneous regression of HCC has rarely been reported (13). The clinical diagnosis criteria for HCC include the presence of elevated AFP levels and the typical imaging characteristics of primary liver cancer including hepatitis and liver cirrhosis (12). The course of spontaneous regression of HCC is difficult to predict. Pathological evidence was obtained only in a few previous cases of spontaneous regression of primary liver cancer, and most cases only met the clinical diagnosis criteria (13). The current patient met the clinical diagnostic criteria for HCC. The reasons for spontaneous regression of liver cancer remain unclear; however, there are several possible explanations.

Previous reports indicate multiple potential mechanisms that may be involved in spontaneous regression of HCC. One is blockage of tumor’s blood supply. Tumor tissues are very sensitive to ischemia, which can result in tumor necrosis and regression (14, 15). This mechanism is also extensively utilized in tumor treatment; for example, sorafenib can inhibit angiogenesis, and transarterial chemoembolization can block the arterial blood supply, thus killing tumor cells (16, 17). In this patient, in the first enhanced CT, the tumor exhibited insignificant enhancement in the vein stage, with portal vein tumor thrombus formation, indicating possible ischemia in the tumor. Thus, a lack of blood supply should be considered a possible cause of spontaneous tumor regression in the current case. Another possible cause of spontaneous regression of liver cancer is the immune response. In previous reports of spontaneous HCC regression, interleukin (IL) 18, IL2, IL6, IL12, interferon *γ*, tumor necrosis factor, and natural killer cells were all significantly increased. Of these, tumor necrosis factor may play an important role in spontaneous regression of HCC (18, 19). In another case, Kimura et al. (20) reported that PD-L1 expression was restricted to the area of well-differentiated HCC, while coagulative necrosis areas of the tumor were PD-L1-negative. This observation indicated that the spontaneous regression might have been associated with an ongoing immune response.

In conclusion, herein we have described a rare case of complete spontaneous regression of an HCC tumor without any intervention. There are currently still many unknown factors involved in spontaneous regression of HCC, for example, the specific reason for spontaneous regression, the final outcomes in this group of patients, and whether new tumor foci will occur after partial or complete regression. In our patient, we did not biopsy the original tumor, however, so we could not utilize immunohistochemistry, single-cell sequencing, or spatial transcriptomics to further investigate the mechanism of spontaneous regression. Over the past few decades, much importance has been placed on tumor pathogenesis, but investigating the mechanisms involved in spontaneous tumor regression may be a promising approach with respect to the development of new cancer therapies.

## Data Availability

The original contributions presented in the study are included in the article Supplementary Material, and further inquiries can be directed to the corresponding author.
