# Supplementary material for: Occurrence–regression–recurrence of hepatocellular carcinoma without any intervention: A case report
Source: Front Surg. 2022 Sep 12;9:972446. doi: 10.3389/fsurg.2022.972446 (PMC9632955; doi:10.3389/fsurg.2022.972446)
Supplement: Supplementary file 1 [file Table1.docx]

Supplementary Material

**Table 1: Partial laboratory examination on May 3, 2019.**

| **Variables** | **Results** | **Units** | **Reference** |
| --- | --- | --- | --- |
| **Blood routine:** | | | |
| White blood cells | 2.63 | 10E9/L | 3.50-9.50 |
| Neutrophils | 1.65 | 10E9/L | 1.40-7.10 |
| Lymphocytes | 0.64 | 10E9/L | 0.70-4.75 |
| Monocytes | 0.21 | 10E9/L | 0.10-0.60 |
| Eosinophils | 0.11 | 10E9/L | 0.02-0.52 |
| Basophil | 0.02 | 10E9/L | 0.00-0.06 |
| Neutrophil ratio | 62.74 | % | 40.00-75.00 |
| Lymphocyte ratio | 24.33 | % | 20.00-50.00 |
| Monocyte ratio | 7.98 | % | 3.00-10.00 |
| Eosinophil ratio | 4.18 | % | 0.40-8.00 |
| Basophil ratio | 0.76 | % | 0.00-1.00 |
| Red blood cells | 3.54 | 10E12/L | 4.30-5.80 |
| Hemoglobin | 112 | g/L | 130-175 |
| Hematocrit | 0.41 | **-** | 0.400-0.500 |
| Mean corpuscular volume | 91.22 | fl | 82.00-100.00 |
| Mean corpuscular hemoglobin | 28.60 | pg | 27.00-34.00 |
| Mean corpuscular-hemoglobin concentration | 324.0 | g/L | 316-354 |
| Platelets | 33 | 10E9/L | 100-300 |
| **Liver function:** | | | |
| Aspartate aminotransferase | 95 | U/L | 15-40 |
| Alanine aminotransferase | 41 | U/L | 9-50 |
| Alkaline phosphatase | 105 | U/L | 45-125 |
| Glutamyl transpeptidase | 228 | U/L | 10-60 |
| Alpha-l-fucosidase | 43 | U/L | 0-40 |
| Total bilirubin | 37.3 | μmol/L | 1.7-26.0 |
| Direct bilirubin | 13.7 | μmol/L | 0.0-7.0 |
| Indirect bilirubin | 23.6 | μmol/L | 1.7-17.0 |
| Total protein | 68.0 | g/L | 65.0-85.0 |
| Albumin | 34.2 | g/L | 40.0-50.0 |
| Globin | 33.8 | g/L | 20.0-40.0 |
| Albumin/Globin | 1.01 | **-** | 1.20-2.41 |

**Table 2: Partial laboratory examination on July 23, 2019.**

| **Variables** | **Results** | **Units** | **Reference** |
| --- | --- | --- | --- |
| **Blood routine:** | | | |
| White blood cells | 2.87 | 10E9/L | 3.50-9.50 |
| Neutrophils | 1.88 | 10E9/L | 1.40-7.10 |
| Lymphocytes | 0.59 | 10E9/L | 0.70-4.75 |
| Monocytes | 0.28 | 10E9/L | 0.10-0.60 |
| Eosinophils | 0.10 | 10E9/L | 0.02-0.52 |
| Basophil | 0.02 | 10E9/L | 0.00-0.06 |
| Neutrophil ratio | 65.40 | % | 40.00-75.00 |
| Lymphocyte ratio | 20.60 | % | 20.00-50.00 |
| Monocyte ratio | 9.80 | % | 3.00-10.00 |
| Eosinophil ratio | 3.50 | % | 0.40-8.00 |
| Basophil ratio | 0.70 | % | 0.00-1.00 |
| Red blood cells | 4.40 | 10E12/L | 4.30-5.80 |
| Hemoglobin | 143 | g/L | 130-175 |
| Hematocrit | 0.432 | **-** | 0.400-0.500 |
| Mean corpuscular volume | 98.20 | fl | 82.00-100.00 |
| Mean corpuscular hemoglobin | 32.50 | pg | 27.00-34.00 |
| Mean corpuscular-hemoglobin concentration | 331.0 | g/L | 316-354 |
| Platelets | 37 | 10E9/L | 100-300 |
| **Liver function:** | | | |
| Aspartate aminotransferase | 47 | U/L | 15-40 |
| Alanine aminotransferase | 49 | U/L | 9-50 |
| Alkaline phosphatase | 91 | U/L | 45-125 |
| Glutamyl transpeptidase | 130 | U/L | 10-60 |
| Alpha-l-fucosidase | 31 | U/L | 0-40 |
| Total bilirubin | 27.5 | μmol/L | 1.7-26.0 |
| Direct bilirubin | 6.9 | μmol/L | 0.0-7.0 |
| Indirect bilirubin | 20.6 | μmol/L | 1.7-17.0 |
| Total protein | 72.1 | g/L | 65.0-85.0 |
| Albumin | 40.9 | g/L | 40.0-50.0 |
| Globin | 31.2 | g/L | 20.0-40.0 |
| Albumin/Globin | 1.31 | **-** | 1.20-2.41 |

**Table 3: Partial laboratory examination on January 11, 2021.**

| **Variables** | **Results** | **Units** | **Reference** |
| --- | --- | --- | --- |
| **Blood routine:** | | | |
| White blood cells | 3.17 | 10E9/L | 3.50-9.50 |
| Neutrophils | 2.24 | 10E9/L | 1.40-7.10 |
| Lymphocytes | 0.56 | 10E9/L | 0.70-4.75 |
| Monocytes | 0.31 | 10E9/L | 0.10-0.60 |
| Eosinophils | 0.05 | 10E9/L | 0.02-0.52 |
| Basophil | 0.01 | 10E9/L | 0.00-0.06 |
| Neutrophil ratio | 70.70 | % | 40.00-75.00 |
| Lymphocyte ratio | 17.80 | % | 20.00-50.00 |
| Monocyte ratio | 9.70 | % | 3.00-10.00 |
| Eosinophil ratio | 1.40 | % | 0.40-8.00 |
| Basophil ratio | 0.40 | % | 0.00-1.00 |
| Red blood cells | 4.74 | 10E12/L | 4.30-5.80 |
| Hemoglobin | 157 | g/L | 130-175 |
| Hematocrit | 0.473 | **-** | 0.400-0.500 |
| Mean corpuscular volume | 99.70 | fl | 82.00-100.00 |
| Mean corpuscular hemoglobin | 33.10 | pg | 27.00-34.00 |
| Mean corpuscular-hemoglobin concentration | 332.0 | g/L | 316-354 |
| Platelets | 31 | 10E9/L | 100-300 |
| **Liver function:** | | | |
| Aspartate aminotransferase | 37 | U/L | 15-40 |
| Alanine aminotransferase | 26 | U/L | 9-50 |
| Alkaline phosphatase | 67 | U/L | 45-125 |
| Glutamyl transpeptidase | 49 | U/L | 10-60 |
| Alpha-l-fucosidase | 35.3 | U/L | 0-40 |
| Total bilirubin | 33.3 | μmol/L | 1.7-26.0 |
| Direct bilirubin | 11.7 | μmol/L | 0.0-7.0 |
| Indirect bilirubin | 21.6 | μmol/L | 1.7-17.0 |
| Total protein | 75.0 | g/L | 65.0-85.0 |
| Albumin | 46.2 | g/L | 40.0-50.0 |
| Globin | 28.8 | g/L | 20.0-40.0 |
| Albumin/Globin | 1.60 | **-** | 1.20-2.41 |
